# Global burden of intellectual disability attributable to preterm birth from 1990 to 2021 and projections

**DOI:** 10.1016/j.isci.2026.117114

**Published:** 2026-08-06

**Authors:** Qishuai Zhuang, Qiyao Zhang, Chunhua Yang, Min Liu, Xiaobing Li, Yue Wang, Fengchun Gao

**Affiliations:** 1 Medical Research Center, Jinan Maternity and Child Care Hospital Affiliated to Shandong First Medical University, Jinan 250000, China; 2 Shandong Institute of Brain Science and Brain-inspired Research, Shandong First Medical University and Shandong Academy of Medical Sciences, Jinan 250117, China

**Keywords:** preterm birth, intellectual disability, global burden, inequality, prediction

## Abstract

This study comprehensively quantified intellectual disability (ID) global burden attributable to preterm birth, utilizing data from Global Burden of Disease (GBD) 2021. In 2021, global prevalence declined with age, and males exhibited higher burden than females across nearly all age groups. Over the past three decades, prevalence of preterm birth-resulted ID increased. Substantial variation was observed across countries, socio-demographic index (SDI) levels, and severity categories. Higher burden was concentrated on low/middle-SDI countries, although the inequality has shown a slight decreasing trend. Population growth and aging emerged as major drivers of increasing burden. Projections suggest that by 2031, prevalence will decrease mainly among severe ID population, while a slight increase is expected among females with mild forms of ID. These results underscore the urgent need for targeted, equity-focused prevention and intervention strategies, especially in low/middle-SDI regions, to mitigate this growing public health concern.

## Highlights

• Global prevalence declined with age, and males exhibited higher burden than females in 2021
• Prevalence of preterm birth-resulted ID increased over the past three decades
• Low-SDI countries experienced significantly higher burden
• Prevalence will decrease mainly among severe ID population in the future prediction trend

## Graphical abstract

**Figure:**
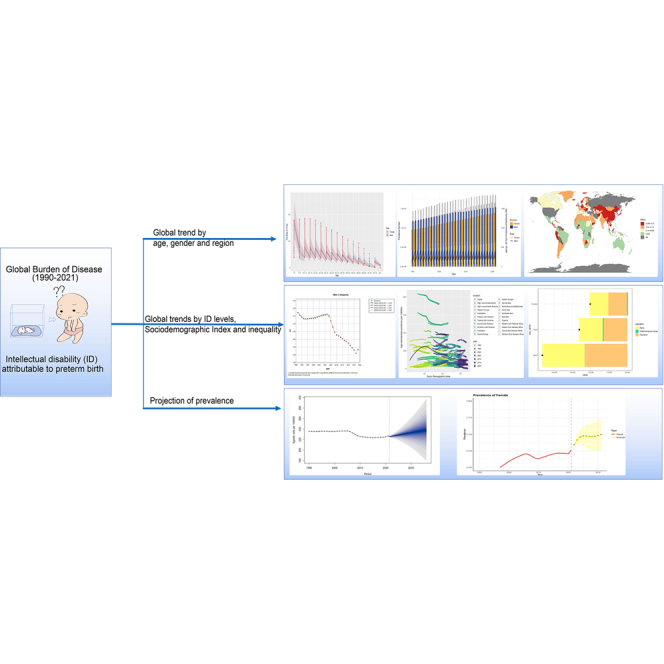

## Teaser

Health sciences; Medicine; Medical specialty; Pediatrics; Public health

## Introduction

Preterm birth, defined as delivery before 37 completed weeks of gestation, remains a major global health concern and accounts for approximately 10% of all live births worldwide.^1^ Recent estimates indicate that nearly 15 million infants are born preterm annually, and more than one million neonatal deaths are directly attributable to complications of prematurity.^2^ Advances in neonatal care, including antenatal corticosteroids, surfactant replacement therapy, and advanced respiratory support, have substantially improved survival among very preterm (<32 weeks) and extremely preterm (<28 weeks) infants.^3^^,^^4^ As survival has increased, greater attention has been directed toward the long-term consequences of prematurity, particularly neurodevelopmental outcomes such as intellectual disability (ID), which often persist into adolescence and adulthood.

ID is recognized as a common neurodevelopmental disorder, characterized by significant limitations in both intellectual functioning and adaptive skills. Intellectual functioning points to general mental capability including learning, reasoning, problem resolving, evaluated by intelligence quotient (IQ) test to classify its severity. Adaptive skills refer to conceptual, social, and practical skills learned and practiced in daily life.^5^ ID for preterm infants leads to adverse outcomes at school age and adolescence.^6^^,^^7^^,^^8^ Research consistently demonstrates that children born preterm achieve lower scores on IQ tests than their term-born peers, a gap that persists into adolescence and adulthood.^9^ Moreover, the severity of impairments tends to increase with decreasing gestational age. However, conventional IQ-based assessments may not fully capture more subtle deficits in executive function, attention regulation, and socioemotional processing, all of which contribute substantially to long-term functioning.

Addressing the intellectual consequences of preterm birth is of paramount significance to clinical practice, healthcare planning, and public health policy. ID not only diminishes quality of life for affected individuals but also imposes considerable social and economic burdens on families, healthcare systems, and society. Accurate quantification of this burden is therefore critical for informing prevention strategies, optimizing resource allocation, and guiding policy development.

Despite extensive research on neurodevelopmental outcomes after preterm birth, several critical gaps remain. First, most studies have focused on early childhood outcomes, whereas longitudinal evidence extending into adolescence and adulthood remains scarce.^10^^,^^11^ This lack of long-term data limits the ability to identify key developmental milestones or critical windows for intervention. Second, traditional measures, such as IQ scales, are often inadequate for capturing subtle intellectual deficits or more complex neuropsychological impairments.^12^ Third, substantial geographical disparities exist in the reported prevalence and severity of ID, driven by divergence in neonatal care practices, healthcare accessibility, and socioeconomic conditions.^13^ These disparities underscore the need for region-specific data to better inform targeted interventions and policies.

Systematic collation of available data and periodic estimates of the prevalence of preterm-related ID using comparable methods are needed to assess the burden at global, regional, and national levels, and to understand how the burden is changing over time. Regular estimates are also needed to raise awareness of preterm birth as an important global public health problem, to inform the implementation or redesign of health policies and programs, and guide resource allocation. These estimates can also be used to monitor the effect of such interventions.

The Global Burden of Disease (GBD) 2021 dataset provides an invaluable opportunity to comprehensively assess the worldwide burden of ID attributed to preterm birth across different regions, age groups, and time periods. Unlike the study by Wang et al., which focused on the ID burden of preterm birth during childhood and adolescence,^14^ the present study leveraged GBD data to evaluate global and regional disparities and risk drivers in years lived with disability (YLDs) and prevalence for ID caused by preterm birth, highlighting ID classification at all ages using a range of analytical and statistical strategies. Importantly, we introduced complementary forecasting approaches to comparatively analyze future burdens over the coming decade, accounting for advancements in neonatal care, demographic trends, and healthcare disparities.

## Results

### Global prevalence and YLDs trends of ID attributable to preterm birth by gender and age group

**Figure 1:**
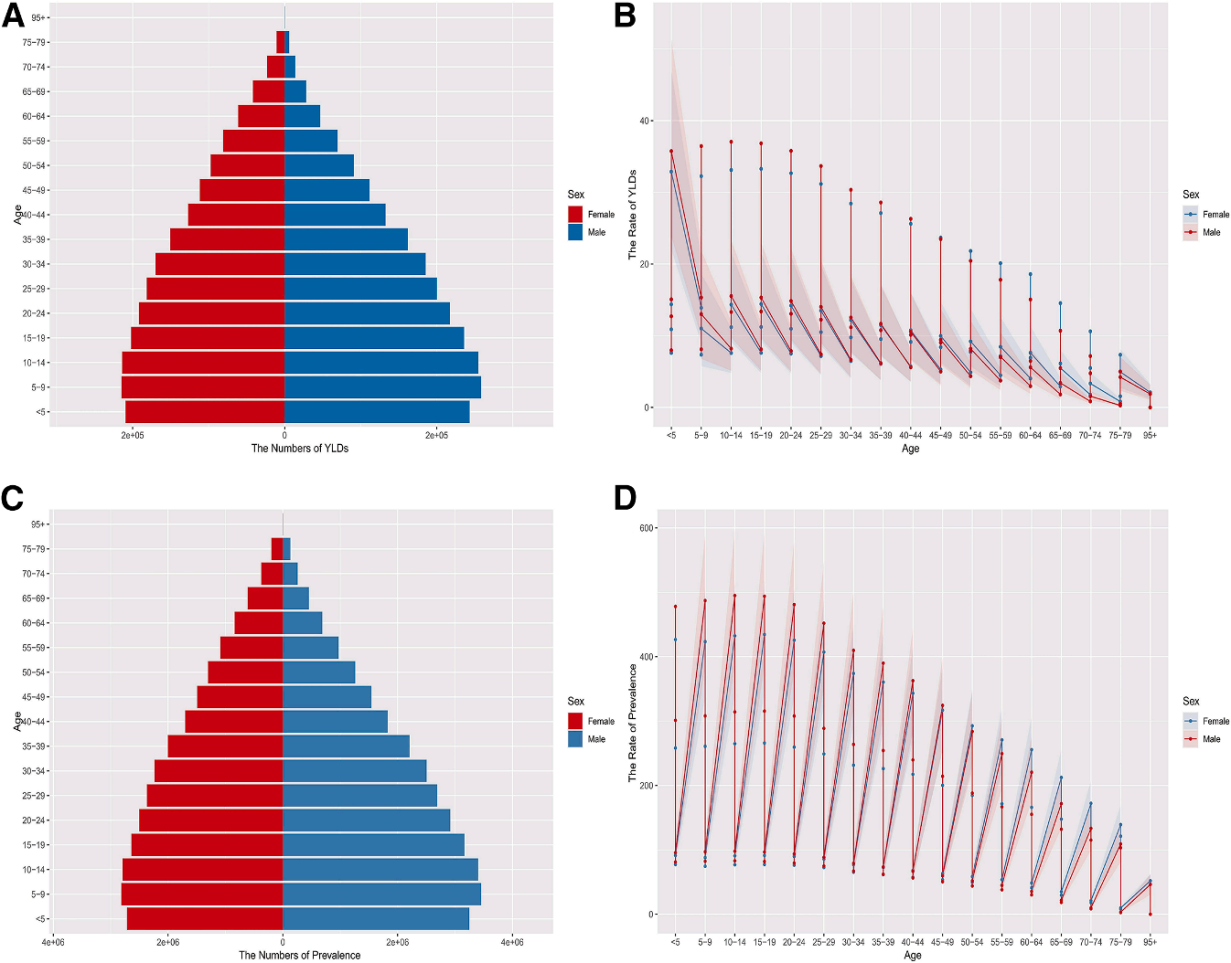
Age-specific number and rates of YLDs and prevalence of ID ascribed to preterm birth by gender in 2021 (A) Comparison of YLDs number between females and males across different age groups in 2021. (B) Comparison of YLDs rates between females and males across different age groups in 2021. (C) Comparison of prevalence number between females and males across different age groups in 2021. (D) Comparison of prevalence rates between females and males across different age groups in 2021. Abbreviations: YLDs, years lived with disability; ID, intellectual disability.

In 2021, ID attributable to premature birth were more common among people aged 5 to 14, and subsequently the global prevalence and YLDs counts and the corresponding rates gradually decreased with age (Figures 1A–1D). Both males and females followed the same trend, and the prevalence and YLDs number and rates of males were higher than those of females (Figures 1A–1D). From 1990 to 2021, the prevalence rate of this disease was generally on the rise (Figure S1A). The YLDs for both sexes were increasing (Figure S1B).

### Global distributions for prevalence and YLDs of ID attributable to preterm birth

**Figure 2:**
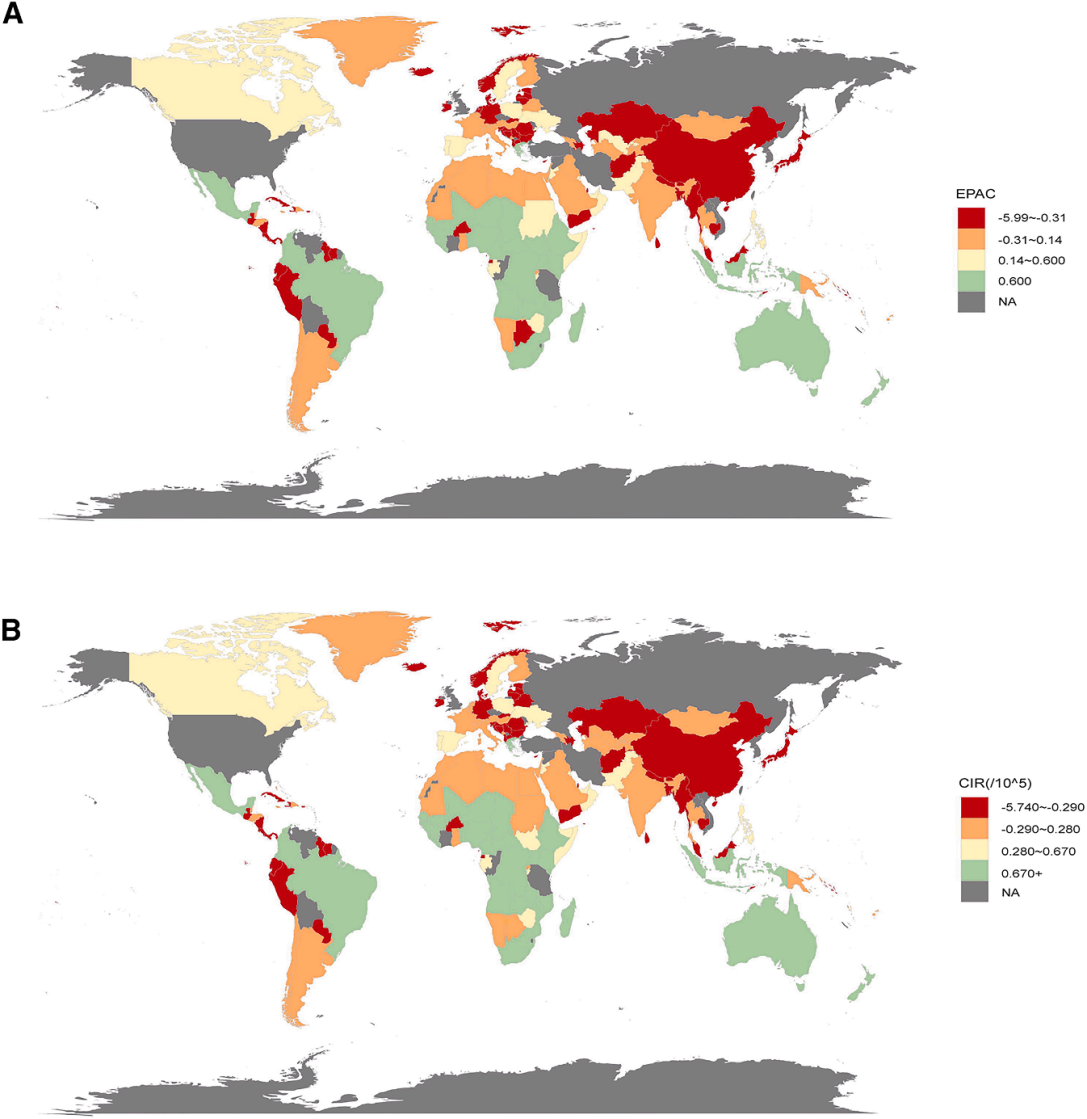
Global maps of prevalence and YLDs in 204 countries and territories (A) Prevalence. (B) YLDs. Abbreviations: YLDs, years lived with disability.

From 1990 to 2021, the disease burden of premature birth-resulted ID performed noticeable divergences across 204 countries and territories. Among countries and territories, the prevalence of 121 countries or regions represented downward trends, with the most substantial reduction in Republic of Korea. Sixty-seven countries or regions are estimated to be on the rise. Among them, Ethiopia recorded the largest increase, followed by Nigeria. Sixteen countries or regions such as Rwanda, Thailand, and Vanuatu remained stable (Figure 2A). With regard to YLDs, 114 countries or regions reported to downward trend, with the largest decline in Republic of Korea. Eighty-two countries or regions witnessed upward tendencies, with Ethiopia recording the most obvious increase, secondarily Nigeria. Eight countries or regions, namely Saudi Arabia and Belize, maintained stabilized (Figure 2B).

### Global prevalence trends in ID attributable to preterm birth by ID levels and sex

We observed that compared with 1990, the age-standardized prevalence rate in 2021 of premature birth-evoked ID in both sexes in 204 countries and territories were increasing respectively. Concretely, regarding developmental ID in females, the top three countries and regions in terms of increase in quantity were Yemen, India, and Egypt (Figure S2A), which was in accordance with mild ID (Figure S2E), whilst the top three countries and regions in terms of increase rate were Qatar (396%), United Arab Emirates (343%), and Nigeria (305%) (Figure S2B). For males, ranking the top three countries and territories of increment were Yemen, India, Afghanistan (Figure S2C), which coincided with mild ID (Figure S2G), while the top three countries and territories of increase rate were United Arab Emirate, Qatar, and Bahrain (Figure S2D). The top three countries and territories of increase ratio in mild ID for females were Australia (459%), United Arab Emirates (403%), and Qatar (390%) (Figure S2F) whereas them for males were Australia (870%), United Arab Emirate (578%), and Qatar (447%) (Figure S2H). When considering moderate and severe ID, the top three countries and territories were concordant in both aspects. In detail, Saint Lucia, Trinidad and Tobago, and Mauritania took the top-three increment when Nigeria, Qatar, and Angola recorded the top-three increased quotients (Figures S2I–S2P).

### Joinpoint regression analysis for prevalence by genders and ID levels

**Figure 3:**
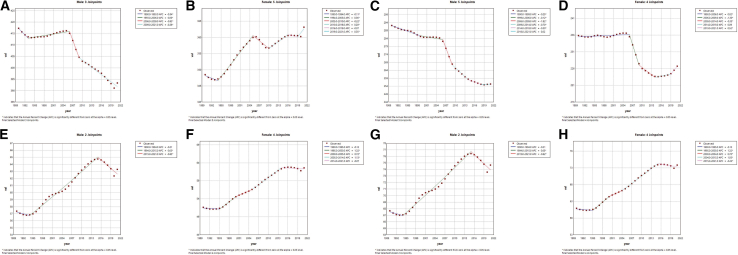
Joinpoint regression analysis trends in age-standardized prevalence rate among developmental and its subcategory ID levels, 1992–2021 (A) Trends in age-standardized prevalence rate in males developmental ID group. (B) Trends in age-standardized prevalence rate in females developmental ID group. (C) Trends in age-standardized prevalence rate in males mild ID group. (D) Trends in age-standardized prevalence rate in females mild ID group. (E) Trends in age-standardized prevalence rate in males moderate ID group. (F) Trends in age-standardized prevalence rate in females moderate ID group. (G) Trends in age-standardized prevalence rate in males severe ID group. (H) Trends in age-standardized prevalence rate in females severe ID group. Abbreviations: ID, intellectual disability.

The Joinpoint regression model was applied to parse the temporal trends of age-standardized prevalence rate in different ID levels, displaying distinct variations in age-standardized prevalence rate trends among ID levels. We found that for developmental ID (Figures 3A and 3B), prevalence in both male and female populations exhibited a trend of an initial descend from 1990 to 1994 (male, APC, −0.34, 95% CI, −0.58 ∼ −0.11; female, −0.11, −0.17 ∼ −0.05, Table S1) and then evident escalate from 1994 to 2005 (male, APC: 0.06, 95% CI, 0.03∼0.08; female, 0.36, 0.34–0.37, Table S1), with a more pronounced spike observed in females. Then, males manifested a consistently significant downward trend while females emerged a trend of first slowing and then increasing, with the division point at year 2000. For mild ID (Figures 3C and 3D), from 1990 to 2006, prevalence in males showed a slow decline whereas it showed a very slight elevation in females, almost no change. Next, prevalence among males showed a sharp decline by 2019, while for females, it experienced a discernible descent from 2006 to 2014 and then observed a slowdown ascent from 2014 to 2019. Subsequently, prevalence for both genders increased from 2019 to 2021. For moderate and severe ID (Figures 3E–3H), prevalence for both sexes performed an initial downward trend from 1990 to 1995, followed by surging from 1995 to 2014 (moderate ID) or 2015 (severe ID), afterward, spanning from 2014 or 2015 to 2021, prevalence for males markedly diminished (APC, −0.63, 95% CI, −0.86 ∼ −0.39, Table S1) while it showed a slight diminution for females (APC, −0.07, 95% CI, −0.19 ∼ −0.04, Table S1).

### Age, period and cohort influences on prevalence and YLDs

Figure S3 in Supplement1 delineates the effects of age, period and cohort on the prevalence and YLDs trends of ID diseases caused by premature birth. The prevalence and YLDs experienced a rapid decrease followed by an uptick at the age of 85 as the boundary (Figures S3A and S3E). For period relative risk (RR) values showed no significant difference from 1994 to 2021 for prevalence, although the trend was to increase first and then decrease. While YLDs manifested a substantial upward trend (Figures S3B and S3F). The cohort RR values for prevalence and YLDs was significantly higher in the early birth cohort, during 1962–1967 at the lowest point, followed by a decrease trend in the latest birth cohort (Figures S3C and S3G). The local drift values for prevalence decreased from 2.148 in the 20–24 age group to −0.362 in the 45–49 age group, and further to −4.027 in the above 95 age group (Figure S3D). Similarly, local drift values for YLDs decreased from 2.447 in the 20–24 age group to −0.275 in the 50–54 age group, and further to −3.996 in the above 95 age group (Figure S3H).

### The co-evolution of age-standardized burden estimates and SDI for ID caused by preterm birth globally and in the GBD region from 1990 to 2021

**Figure 4:**
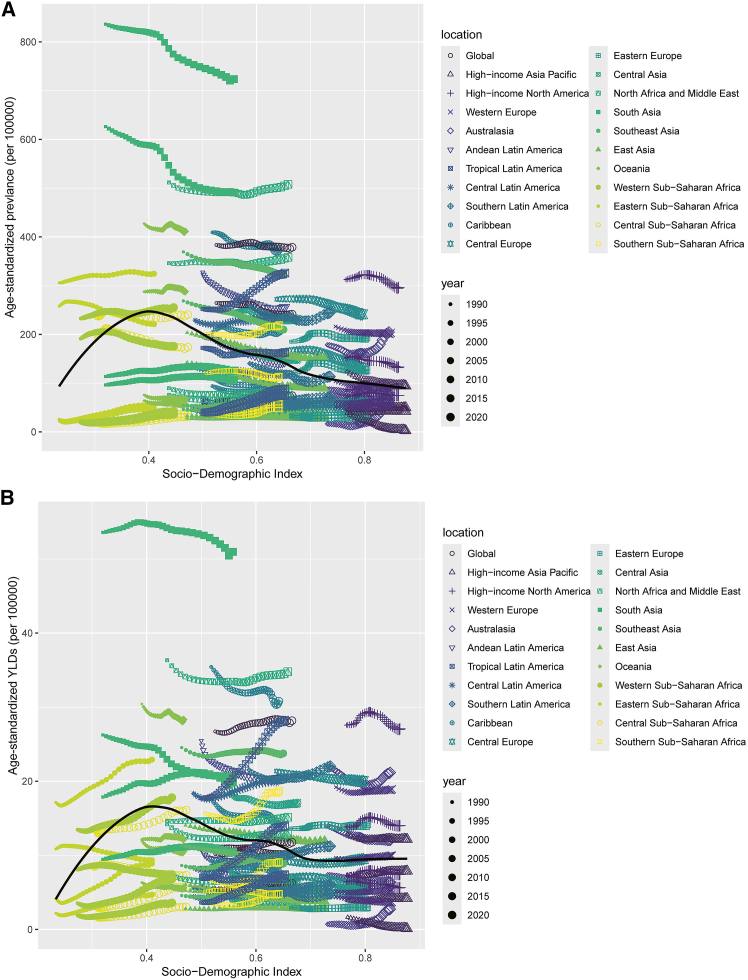
Correlation between SDI and age-standardized prevalence rate and YLDs of ID resulted from preterm birth, 1990–2021 (A) Correlation between SDI and age-standardized prevalence rate of preterm birth-resulted ID, 1990–2021. (B) Correlation between SDI and YLDs of preterm birth-resulted ID, 1990–2021. Abbreviations: SDI, socio-demographic index; YLDs, years lived with disability; ID, intellectual disability.

Higher age-standardized prevalence rate and YLDs of ID resulted from preterm birth were predominantly clustered in low- and middle-SDI countries. Noticeably, with the SDI increases, age-standardized prevalence rate and YLDs initially showed an upward trend and reached a peak, and then gradually declined (Figures 4A and 4B).

### Cross-country inequality of YLDs in ID caused by premature birth

The YLDs of ID attributable to premature birth among countries in 1990 and 2021 present health inequalities closely related to SDI. Specifically, SII and CIX values showed that the higher burden was concentrated in countries and regions with lower SDI. With passage of time, the extent of this health inequality has slightly reduced (Figure S4).

### Decomposition estimates for determinants discrepancies at population-level on prevalence and YLDs of ID due to preterm birth

**Figure 5:**
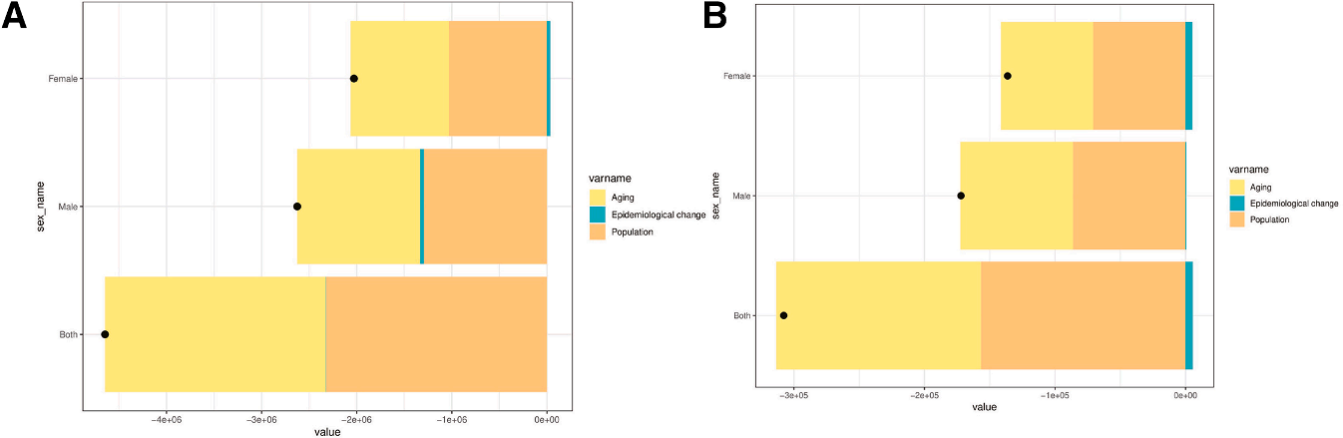
The decomposition of population-level risk factors for the changes in prevalence and YLDs of ID from preterm birth Population-level driving differences in population aging, epidemiological change and population growth for prevalence (A) and YLDs (B) of ID from preterm birth. Black dots designate the total contribution driven by overall three factors. Positive/negative value for each factor expresses positive/negative force in prevalence and YLDs, correspondingly. Abbreviations: YLDs, years lived with disability; ID, intellectual disability.

Population-level risk factors were decomposed independently. The decomposition estimated the changes in prevalence and YLDs of ID from preterm birth due to risk factors, involving population aging, population growth, and epidemiological change. Aging and population growth pronouncedly contributed to mitigate prevalence and YLDs, while forces of epidemiological change on females and males were opposite (Figure 5).

### Forecasting prevalence from 2021 to 2031

**Figure 6:**
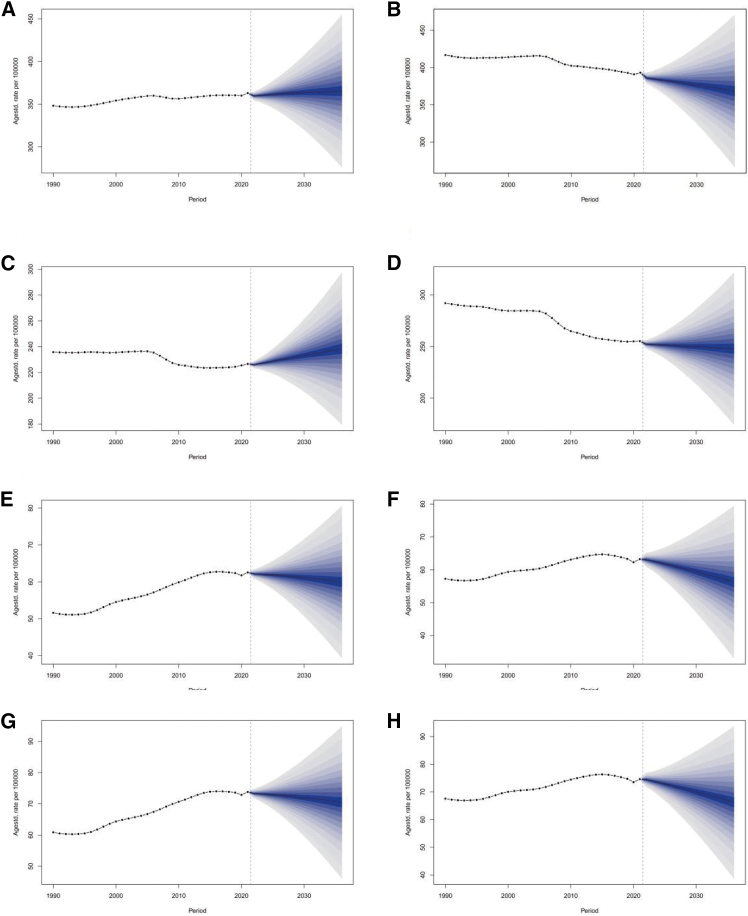
BAPC model predictions for age-standardized prevalence rates on developmental and its subcategory ID levels by premature birth from 2021 to 2031 (A) Predictions for age-standardized prevalence rates in females developmental ID group. (B) Predictions for age-standardized prevalence rates in males developmental ID group. (C) Predictions for age-standardized prevalence rates in females mild ID group. (D) Predictions for age-standardized prevalence rates in males mild ID group. (E) Predictions for age-standardized prevalence rates in females moderate ID group. (F) Predictions for age-standardized prevalence rates in males moderate ID group. (G) Predictions for age-standardized prevalence rates in females severe ID group. (H) Predictions for age-standardized prevalence rates in males severe ID group. Abbreviations: BAPC: Bayesian age-period-cohort; ID, intellectual disability.

Figure 6 depicted Bayesian age-period cohort (BAPC) model forecasts for age-standardized prevalence rates between genders of developmental and its subcategory ID levels by premature birth from 2021 to 2031. Prevalence for females with developmental and mild ID, continues to rise, just increase of developmental ID is relatively slight, while projected decrease of it for males (Figures 6A–6D). With ID severity, both moderate and severe ID, prevalence for females and males is expected to be lessened, but the extent of the decrease varies (Figures 6E–6H).

We further used auto-regressive integrated moving average (ARIMA) model to predict the prevalence of premature birth-resulted developmental and its subcategory ID levels from 2021 to 2031. For developmental, moderate and severe ID, prevalence for both females and males may ascent with passage of time (Figures S5A, S5B, and S5E–S5H). Noticeably, for mild ID, prevalence for females may increase slightly yearly while prevalence for males will decline (Figures S5C and S5D). The above data highlights further burden will rise in almost all ID from premature birth, apart from males mild ID. It is worth noting that the projected tendencies from the two models are not completely accordant, which could be due to the fact that the prevalence of preterm birth resulted-ID fluctuated significantly from 1990 to 2021.

In order to better evaluate the prediction accuracy of the two models for the prevalence rate, we estimated the fitting situation of these two models by calculating the MAE, MSE, and MAPE and found that the prediction accuracy of BAPC (e.g., MAPE at 0.01%–0.09%) was significantly better than that of ARIMA (e.g., MAPE at 1.4%–5.5%), and the specific prediction values were provided in the Table 1.

**Table 1:** Applying MAE, MSE, and MAPE values to estimate BAPC and ARIMA prediction accuracy for further prevalences on female and male developmental and its subcategory ID by premature birth

BAPC	ID classification	MAE	MSE	MAPE (%)
Female	Developmental ID	3.84E−05	1.18E−10	1.18
	Mild ID	5.64E−05	2.15E−10	2.89
	Moderate ID	1.92E−05	4.10E−07	3.23
	Severe ID	2.72E−05	1.14E−06	3.81
Male	Developmental ID	2.18E−04	1.14E−06	3.12
	Mild ID	2.23E−04	5.23E−08	8.70
	Moderate ID	2.13E−05	5.20E−10	3.40
	Severe ID	1.06E−05	1.50E−07	1.42

**ARIMA**				

Female	Developmental ID	2.49E−02	6.28E−04	3.46
	Mild ID	8.33E−03	7.19E−05	1.86
	Moderate ID	6.98E−03	4.99E−05	5.51
	Severe ID	6.86E−03	4.70E−05	4.64
Male	Developmental ID	1.13E−02	1.50E−04	1.42
	Mild ID	1.37E−02	1.94E−04	2.66
	Moderate ID	1.95E−03	4.85E−06	1.52
	Severe ID	2.30E−03	7.09E−06	1.52

Abbreviations: MAE, mean absolute error; MSE, mean square error; MAPE, mean absolute percentage error; BAPC, Bayesian age-period cohort; ARIMA, auto-regressive integrated moving average; ID, intellectual disability.

To more rigorously evaluate model robustness, we performed time-series cross-validation (rolling forecast) on both the BAPC and ARIMA models. The cross-validation errors aligned well with those obtained from the previous test set (2021), with the BAPC model exhibiting markedly lower errors than the ARIMA model across all subgroups (gender and ID levels). Mean MAPE values ranged from 0.01% to 0.1% for BAPC, versus 1.5%–5.5% for ARIMA (Table 2). These results further underscore the superior robustness of the BAPC model in forecasting the prevalence of cognitive disorders attributable to preterm birth.

**Table 2:** Comparison of rolling cross-validation errors between BAPC and ARIMA models

BAPC	ID classification	MAE	MSE	MAPE (%)
Female	Developmental ID	1.25E−10	3.92E−05	0.01
	Mild ID	2.18E−10	5.68E−05	0.03
	Moderate ID	4.15E−07	1.95E−05	0.03
	Severe ID	1.16E−06	2.75E−05	0.04
Male	Developmental ID	1.15E−06	2.19E−04	0.03
	Mild ID	5.30E−08	2.25E−04	0.09
	Moderate ID	5.25E−10	2.15E−05	0.03
	Severe ID	1.55E−07	1.08E−05	0.01

**ARIMA**				

Female	Developmental ID	6.41E−04	2.51E−02	3.48
	Mild ID	7.25E−05	8.50E−03	1.88
	Moderate ID	5.01E−05	7.10E−03	5.54
	Severe ID	4.85E−05	6.90E−03	4.63
Male	Developmental ID	1.52E−04	1.14E−02	1.43
	Mild ID	1.98E−04	1.39E−02	2.68
	Moderate ID	4.92E−06	1.98E−03	1.53
	Severe ID	7.25E−06	2.35E−03	1.51

Abbreviations: MAE, mean absolute error; MSE, mean square error; MAPE, mean absolute percentage error; BAPC, Bayesian age-period cohort; ARIMA, auto-regressive integrated moving average; ID, intellectual disability.

## Discussion

This study presents the first systematic global appraisal, utilizing 2021 GBD data, of prevalence and YLDs of premature birth-related ID across different subtypes, segmented by all age groups, gender, year, period, cohort, SDI levels, and geographic regions, together with forecasts of future trends. The temporal patterns of prevalence varied according to ID severity. Higher burden was concentrated in low- and middle-SDI countries. Cross-country inequalities in disease burden between poor and rich nations persisted. Decomposition analysis of population-level determinants revealed that aging and population growth alleviated the disease burden. The BAPC and ARIMA models yielded divergent forecasts varying by ID levels, thereby offering actionable opportunities for improvement across all levels of preterm-related ID.

Amenable to modern neonatal and pediatric care, more than 95% of preterm survivors worldwide survive into adulthood.^15^^,^^16^ Among school-aged children and adolescents born preterm, 35%–50% are diagnosed with neurodevelopmental dysfunctions that require special educational support.^17^ Our analysis shows that since 1990, global prevalence and YLDs of preterm-related ID have increased for both sexes—a concerning upward trajectory that underscores a growing public health challenge. Both prevalence and YLDs peaked in children aged 5–14 years and then progressively declined in older age groups, and the burden was consistently higher in males than in females. Previous studies have shown that neurodevelopmental disorders affect approximately one million preterm survivors born annually and may persist into adulthood.^18^^,^^19^^,^^20^ A Finnish birth-cohort study identifies that the cumulative incidence of ID among male children is 1.43-fold that among females, and the risk of delayed development, postponed school start, or enrollment in special education programs is two to three times higher in boys than in girls.^21^ Several studies have reported greater biological vulnerability in preterm males, leading to a higher risk of adverse neurodevelopmental outcome.^22^^,^^23^^,^^24^ Magnetic resonance imaging data have suggested that male infants are particularly vulnerable to the detrimental effects of preterm birth on white matter development, possibly owing to differential resistance to hypoxia.^25^^,^^26^

Hudson et al. have observed distinct methylation patterns between male and female preterm children, with one of the most significantly differentially methylated regions located in *NAB1*, a gene involved in modulating neuronal differentiation and gene expression.^27^^,^^28^ As reviewed by Ardalan et al., neurodevelopmental disorders in preterm infants generally exhibit sexual dimorphism, and the etiology is multifactorial—likely involving immune activation in the brain, complex interactions among sex hormones, brain genetics, glial cell activation, and cytokine induction.^29^ In summary, sex appears to shape the mechanisms by which the developing brain is susceptible to preterm birth, although the precise neurobiological mechanisms remain to be elucidated.

We observed substantial heterogeneity in prevalence, YLDs, and age-standardized prevalence rates across 204 countries and territories. Although more than half of the nations showed declining burdens, pronounced increases were noted in several countries, including Ethiopia, Nigeria, and Yemen. Countries with dramatic increases in the prevalence of preterm-related ID, especially in terms of dysgnosia severity level, were Yemen, India, Afghanistan, Egypt, Nigeria, Niger, Angola, Saint Lucia, Trinidad and Tobago, and Mauritania, with already existing high preterm birth rates. Previous epidemiological estimates from 103 countries have similarly indicated that gaps or weaknesses in national routine data on preterm birth are most evident in low- and middle-income countries, particularly in sub-Saharan Africa and southern Asia.^1^ Notably, we found that extremely high percentage increases (300%–870%) in the prevalence of mild ID in high-resource settings such as Australia and Gulf Cooperation Council states. An Australia cohort study has exhibited that rate of mild intellectual impairment at age 8 years is higher in the 1997 cohort than in the 1991–1992 cohort.^30^ Another Australia cohort study has suggested that academic achievement scores at age 8 years among children born extremely preterm are lower in 2005 than in 1997, while the proportion experiencing academic problems are higher in 2005 than in other earlier periods.^31^ Together, these data indicate an uneven distribution of preterm-related ID burden across world regions.

Joinpoint and APC analyses provided further insights into temporal patterns. From our analysis, for mild ID, the burden before 2006 declined significantly in males but remained virtually unchanged in females; between 2014 and 2019, the burden decreased markedly in males while showing a slight upward trend in females. For moderate and severe ID, both sexes exhibited an increasing historical trend from 1995 to 2015, followed by a decline-more pronounced in females. These findings suggest that the sex distribution of preterm-related ID may vary over time, indicating potential period-specific differences. The age effect was characterized by peak burden at birth, a sustained decline up to age 85 years, and then a gradual ascent thereafter. The period effect indicated a steadily increasing population-level burden over time. Importantly, the cohort effect showed a lower burden in recent birth cohorts, suggesting generational progress-although a slight uptick was observed for the 2019–2021 cohort, possibly related to health-care disruptions during the COVID-19 pandemic.^32^ Collectively, these outcomes may suggest certain progress in reducing ID burden from premature infants over time. Nevertheless, persistent sex disparities and residual severe disability burden underscore unmet public health challenges, especially the need for tiered surveillance systems that prioritize moderate-to-severe cases and pandemic-resilient frameworks for preterm infant care.

The relationship between SDI and disease burden followed an inverse-U pattern: age-standardized rates rose with SDI, peaked at middle-SDI levels, and declined in high-SDI regions. This pattern likely reflects competing mechanisms that in low-SDI regions, high neonatal mortality reduces survival of preterm infants, artificially curbing recorded ID burden. As SDI rises, improved neonatal survival may expose more infants to long-term neurodevelopmental sequelae, amplifying burden until advanced healthcare systems in high-SDI regions implement effective preventive and rehabilitative interventions. The disproportionately higher burden of ID attributable to preterm birth in low-/middle-SDI regions may reflect both greater exposures to prematurity and poorer neurodevelopmental outcomes among survivors. Previous GBD analyses have shown that preterm birth incidence, mortality, and DALY rates remain concentrated in resource-limited settings,^33^^,^^34^ where maternal infections, inadequate antenatal care, and adverse socioeconomic conditions increase the risk of premature delivery.^35^^,^^36^^,^^37^ This competing risk of death could lead to an underestimation of the true burden of ID in resource-limited settings. Concurrently, we observed significant cross-national health inequalities in ID burden as measured by YLDs. The concentration of burden in low-SDI settings, as quantified by the SII and CIX, possibly accentuates systemic disparities in perinatal care, early-intervention access, and socioeconomic safety nets.^1^^,^^38^ The modest reduction in inequality between 1990 and 2021 may reflect global efforts such as the United Nations Sustainable Development Goals targeting maternal-child health and poverty reduction.^39^ Nevertheless, persistent disparities highlight unmet needs in low-SDI regions, where limited neonatal intensive care, postnatal follow-up, and intellectual rehabilitation services exacerbate preventable disability.

Decomposition analysis illustrated that population aging and growth exerted mitigating effects on both prevalence and YLDs. A birth cohort study by Kati et al. has revealed that attained lifetime education could alleviate aging-associated neurocognitive impairment, especially among populations born late preterm,^40^ suggesting that expansion of lifelong continuous education may help diminish the burden. Early developmental interventions improve cognitive outcomes in preterm infants during infancy and preschool age,^41^ and population growth may dilute prevalence metrics through higher fertility rates and younger population pyramids, which might account for the protective effect of population growth on this burden.

Currently, it is widely operationalized in field of disease burden prediction by BAPC and ARIMA models. Forecasts using these two models yielded divergent predictions. The ARIMA model predicted rising prevalence for nearly all ID categories, with the sole exception of declining mild ID in males. As a conventional linear approach based on historical time-series analysis, ARIMA has inherent limitations in identifying and forecasting complex nonlinear trends.^42^^,^^43^ It operates on the principle of inertia-a limitation evident from our ARIMA outputs. By consolidating all influencing factors into a single time variable without incorporating external covariates, ARIMA achieves broad applicability, straightforward implementation, and reasonable short-term forecasting accuracy, but it has unavoidable deficiencies in modeling nonlinear data and long-term trends.^44^ Conversely, the BAPC model projected a declining trend across most severity levels and both sexes, except for increasing prevalence of developmental and mild ID among females. The BAPC model explicitly addresses nonlinearity by incorporating prior knowledge and simultaneously modeling the interdependent effects of age, period, and cohort dimensions. This methodological framework enhances predictive capability, robustness, and adaptability, enabling sophisticated analysis of complex data structures and yielding more accurate long-term projections.^45^^,^^46^ The BAPC projection of a sustained decline or slight increase in preterm-related ID prevalence over the next decade is reassuring and likely attributable to improved health-care accessibility, diagnostic and therapeutic advances, and continued health-policy refinements.^47^^,^^48^^,^^49^ However, projected slight increase in mild ID among female preterm infants calls for targeted intervention strategies rather than therapeutic complacency.

In summary, this study provides the first systematic quantification of the influence of multiple risk factors-including age group, sex, year, period, birth cohort, geographic region, SDI, population-level factors, and ID severity-on the global burden of preterm-related ID over a 30-year period, together with forecasts of future trends. Our analysis data indicate several encouraging trends, but disparities persist across multiple dimensions, become of paramount importance to crafting innovative and specialized strategies for prevention and management on addressing this global health challenge.

### Limitations of the study

Although this study leveraged relatively state-of-the-art and robust GBD methodologies, limitations pertain to data precision and modeling refinement should be acknowledged. First, scarcity of original data, especially variations in diagnostic methods across different countries and regions, may bias the analytical results. Second, although we incorporated dimensions including gender, age, location, period, cohort, SDI level and ID grade, the collaborations between these factors and more underlying determinants could not be fully explored. Third, the study relies exclusively on the GBD 2021 dataset, which contains estimates only up to 2021. Access to GBD 2023 data were not granted because of our lack of approved collaborator status. Future studies with access to updated GBD releases should validate whether the patterns observed here persist in more recent years. Fourth, GBD database does not provide individual-level longitudinal data linking preterm birth, survival trajectories, and subsequent ID outcomes, therefore, direct quantification or modeling of survivor bias cannot be achieved in the current research. Finally, more detailed stratified analyses, particularly longitudinal cohort estimates focused on high-burden and high-increase regions, are highly warranted to elucidate specific etiological pathways and to quantify the long-term effectiveness of early interventions.

## Resource availability

### Lead contact

Further information and requests for resources should be directed to and will be fulfilled by the lead contact, Yue Wang (wangyue@sdfmu.edu.cn).

### Materials availability

This study did not generate new unique reagents.

### Data and code availability

• The data used in this study are publicly available from the Global Burden of Disease (GBD) 2021 database, maintained by the Institute for Health Metrics and Evaluation (IHME). All data can be accessed at http://ghdx.healthdata.org/gbd-2021.
• This study does not report original code.
• Any additional information required to reanalyze the data reported in this article is available from the lead contact upon request.

## Acknowledgments

We thank the GBD study 2021 collaborators. This study was funded by the 10.13039/501100001809National Natural Science Foundation of China (10.13039/501100001809NSFC) (82471676), 10.13039/501100007129Natural Science Foundation of Shandong Province (ZR2023MC124, ZR2024QH623, ZR2025LMB019) and Jinan Health High-Caliber Talent Project (202412).

## Author contributions

Y.W. and F.G. conceptualized the idea for the study. Q. Zhuang, Q. Zhang, M.L., and X.L. collected, analyzed, and visualized the data. C.Y. contributed to optimize the data analysis work and wrote the study. Y.W. revised the study. All authors have read the final manuscript and agreed to its publication.

## Declaration of interests

No conflict of interest was declared by the authors.

## STAR★Methods

### Key resources table

**Table:** 

REAGENT or RESOURCE	SOURCE	IDENTIFIER
**Software and algorithms**		

R-4.4.2	The R Foundation for Statistical Computing	https://www.r-project.org/
R Studio 2024.09.1	Posit Software	https://posit.co/downloads/

**Other**		

GBD 2021	IHME	http://ghdx.healthdata.org/gbd-results-tool

### Experimental model and study participant details

All data utilized in this work were assessed from the GBD database.

### Method details

#### Study design and data source

This study conducted a comprehensive analysis of the disease burden of ID caused by preterm birth using data from the 2021 GBD study. The GBD study affords systematic and standardized estimates of disease burden across 204 countries and territories from 1990 to 2021. It applies multiple metrics to quantify the global and regional impact of manifold health conditions, enabling evaluation of trends over time and predicting future burdens. For this study, we focused on preterm birth-related ID and its associated disease burden. Global and regional estimates of YLDs, prevalence, age-standardized prevalence rates, and age-standardized YLDs for IDs caused by preterm birth were extracted for the years 1990–2021. Data were stratified by location, age, sex, and ID severity. Specifically, IDs options for developmental ID and its subcategories including Mild, Moderate ID, Severe ID were chosen.

#### Patient engagement and protocol approvals

This study applied open-source data and carried out in-depth analyses from multiple perspectives without using any personal identification information. Therefore, patient consent was not required.

### Quantification and statistical analysis

Joinpoint regression analysis was utilized to discern variations in annual percentage changes (APCs) with corresponding 95% confidence intervals (CIs) in trends over specific time periods for prevalence rates. Age-period-cohort analysis was conducted to disentangle the influences of age, period, and birth cohort on the burden trend. Decomposition analysis was employed to ascribe burden changes to specific causes, concerning population size, aging, and epidemiological change. To systematically quantify the inequality of YLDs caused by ID in premature birth globally, the slope inequality index (SII) and Concentration Index (CIX) were introduced to construct a comprehensive evaluation system from two dimensions of absolute difference and relative distribution. A positive SII indicates a higher burden in high-SDI countries and a positive CIX represents concentration of burden in high-SDI countries, and vice versa.

We leveraged the Bayesian age-period-cohort (BAPC) and Auto-Regressive Integrated Moving Average (ARIMA) model to predict the age-standardized prevalence for both sexes from 2022 to 2031, respectively. The ARIMA model is a widely used statistical approach for time-series analysis that captures and forecasts trends, seasonality, and stochastic fluctuations in temporal data. As a quantitative method that does not incorporate complex external factors, ARIMA assumes that the target time series can be treated as a predictable stochastic process, enabling effective analysis and future prediction.^42^ Prior to model construction, stationarity and seasonality tests are performed on the raw time series data and model parameters are estimated using maximum likelihood estimation or similar techniques to build model.^50^ In contrast, the BAPC model incorporates the complex interactions of demographic changes over time by integrating age, period, and cohort effects.^45^ Using integrated nested Laplace approximations (INLA) for approximate Bayesian inference, the BAPC model offers a nuanced approach to disease trend prediction, handling data uncertainty and complexity more effectively than traditional models.^51^^,^^52^ In addition, predictive accuracy of both models was evaluated using the mean absolute error (MAE), mean square error (MSE), and mean absolute percentage error (MAPE). Lower values of MAE, MSE, and MAPE indicate higher prediction accuracy. To further validate the robustness of the models, we applied time series cross-validation using a rolling forecast scheme to the BAPC and ARIMA models. Specifically, data from 1990 to 2010 served as the initial training set to predict the values for 2011. Subsequently, the training set was incrementally expanded by adding the actual data of each preceding year, and the models were re-estimated to forecast the next year, iterating through 2020. This process generated point forecasts for the period 2011–2020, from which the prediction errors for each year, MSE, MAE, and MAPE were computed. Finally, the average values of these error metrics were calculated.

All statistical analyses were performed using R software (version 4.1.3) and Joinpoint Regression Program (version 4.9.1.0). *p* < 0.05 signifies statistically significant.
