# Supplementary material for: Global burden of intellectual disability attributable to preterm birth from 1990 to 2021 and projections
Source: iScience. 2026 Aug 6;29(8):117114. doi: 10.1016/j.isci.2026.117114 (PMC13475685; doi:10.1016/j.isci.2026.117114)
Supplement: Supplementary file 1 [file mmc1.pdf]

iScience, Volume *29*

Supplemental information

Global burden of intellectual disability attributable to preterm birth from 1990 to 2021 and projections

Qishuai Zhuang, Qiyao Zhang, Chunhua Yang, Min Liu, Xiaobing Li, Yue Wang, and Fengchun Gao

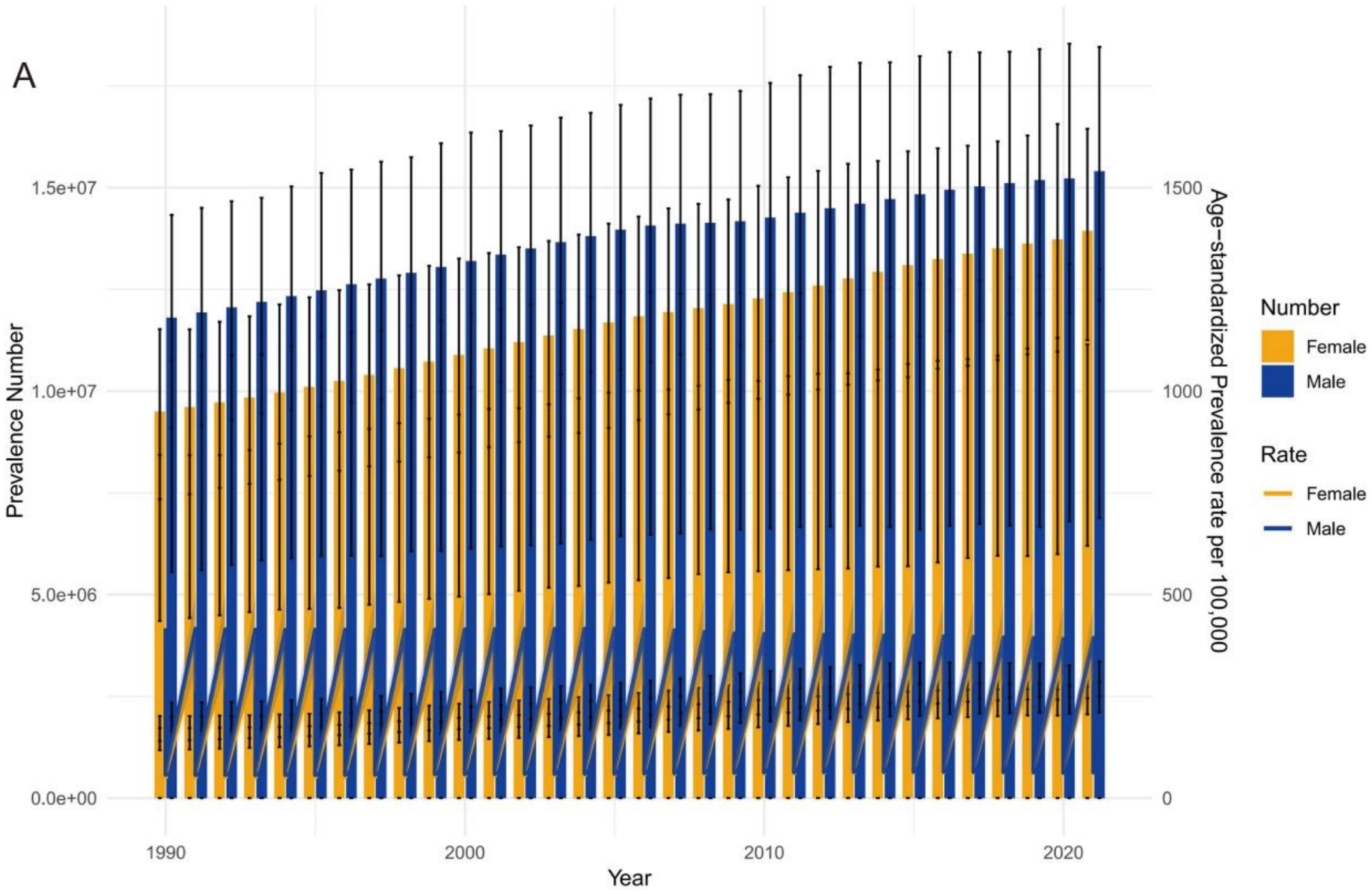

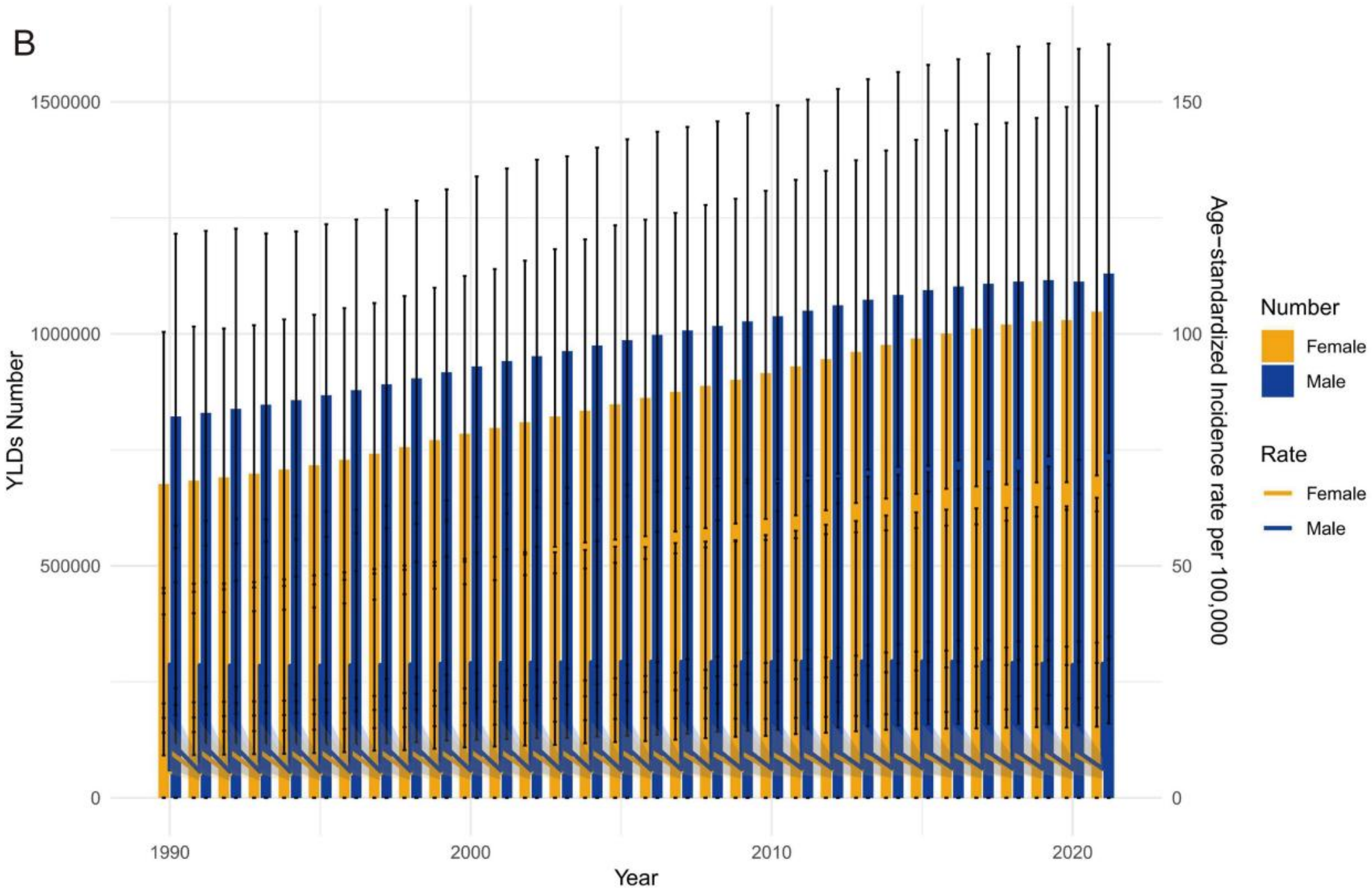

Supplemental Figure S1. Global trends in prevalence and YLDs of ID attributable to preterm birth by sex from 1990 to 2021. A, Global trends in prevalence number of ID attributable to preterm birth by sex from 1990 to 2021. B, Global trends in YLDs number of ID attributable to preterm birth by sex from 1990 to 2021. Abbreviations: YLDs, years lived with disability; ID, intellectual disability.

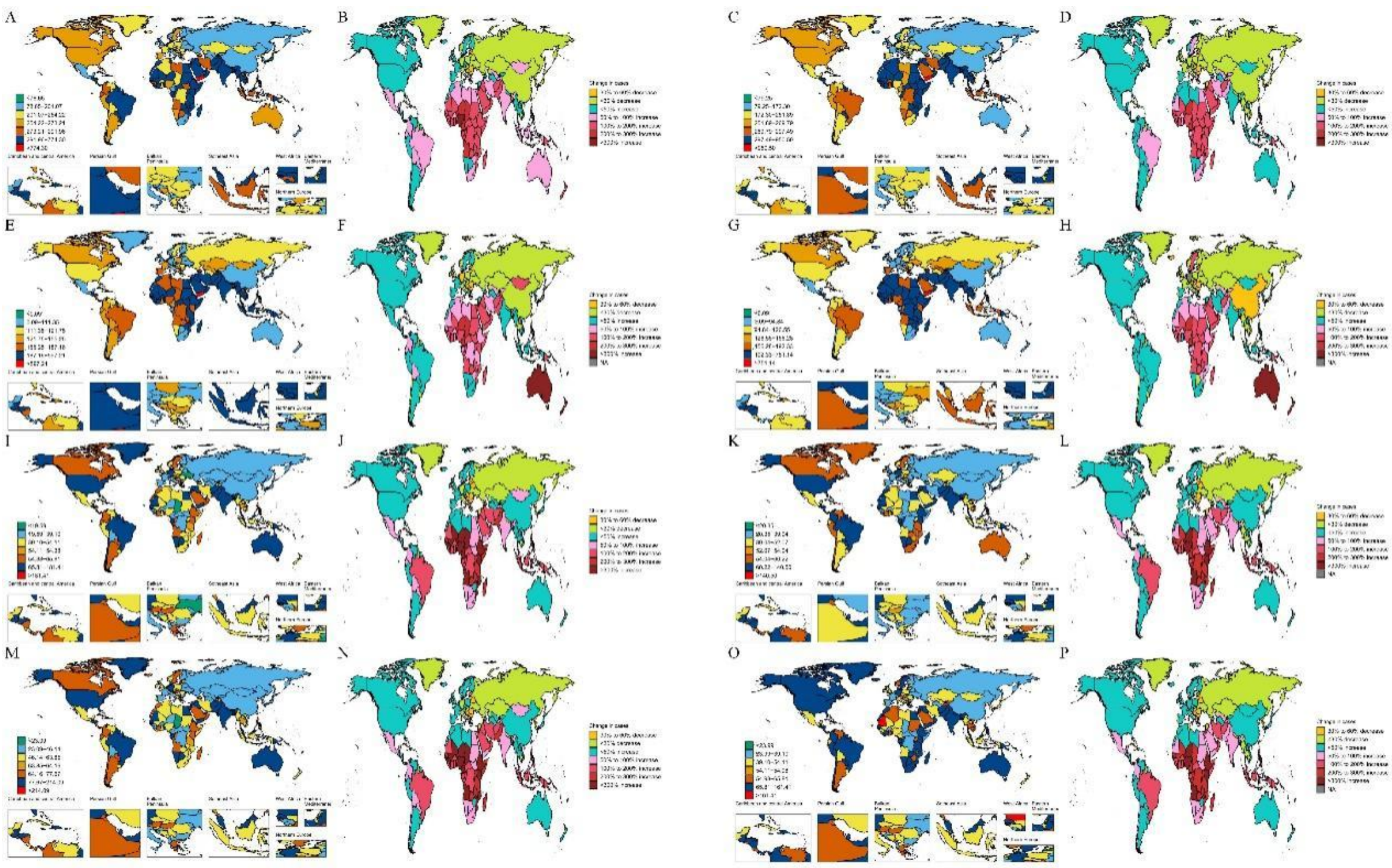

Supplemental Figure S2. Global age-standardized prevalence trends encompassing increment and increase change rate across 204 countries and territories in 2021, in comparison with 2019. A, Global age-standardized prevalence trends of increment of females developmental ID across 204 countries and territories in 2021 compared with 2019. B, Global age-standardized prevalence trends of increment of males developmental ID across 204 countries and territories in 2021 compared with 2019. C, Global age-standardized prevalence trends of increase rate of females developmental ID across 204 countries and territories in 2021 compared with 2019. D, Global age-standardized prevalence trends of increase rate of males developmental ID across 204 countries and territories in 2021 compared with 2019. E, Global age-standardized prevalence trends of increment of females mild ID across 204 countries and territories in 2021 compared with 2019. F, Global age-standardized prevalence trends of increment of males mild ID across 204 countries and territories in 2021 compared with 2019. G, Global age-standardized prevalence trends of increase rate of females mild ID across 204 countries and territories in 2021 compared with 2019. H, Global age-

standardized prevalence trends of increase rate of males mild ID across 204 countries and territories in 2021 compared with 2019. I, Global age-standardized prevalence trends of increment of females moderate ID across 204 countries and territories in 2021 compared with 2019. J, Global age-standardized prevalence trends of increment of males moderate ID across 204 countries and territories in 2021 compared with 2019. K, Global age-standardized prevalence trends of increase rate of females moderate ID across 204 countries and territories in 2021 compared with 2019. L, Global age-standardized prevalence trends of increase rate of males moderate ID across 204 countries and territories in 2021 compared with 2019. M, Global age-standardized prevalence trends of increment of females severe ID across 204 countries and territories in 2021 compared with 2019. N, Global age-standardized prevalence trends of increment of males severe ID across 204 countries and territories in 2021 compared with 2019. O, Global age-standardized prevalence trends of increase rate of females severe ID across 204 countries and territories in 2021 compared with 2019. P, Global age-standardized prevalence trends of increase rate of males severe ID across 204 countries and territories in 2021 compared with 2019. Abbreviations: ID, intellectual disability.

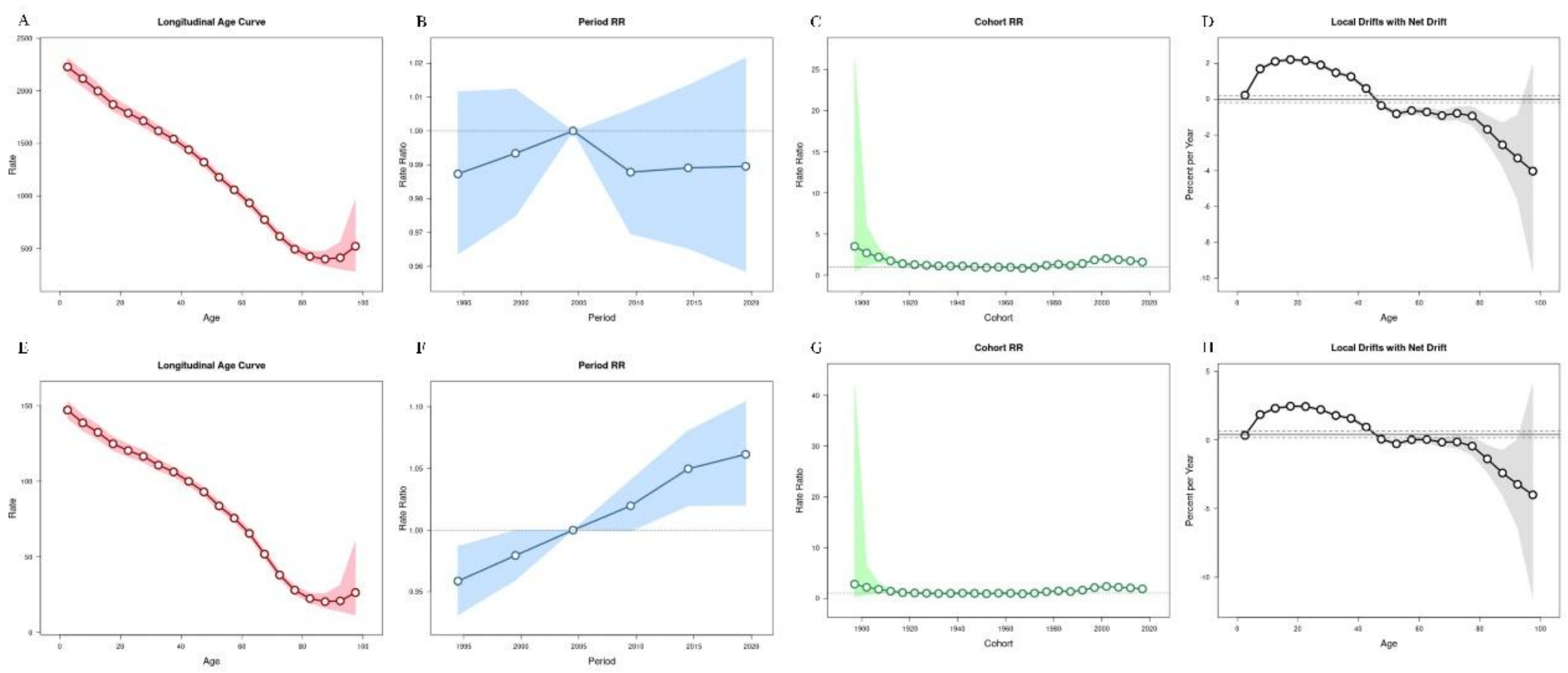

Supplemental Figure S3. Age-period-cohort effects of prevalence and YLDs. A, Age

effects on prevalence. B, Age effects on YLDs. C, Period effects on prevalence. D, Period effects on YLDs. E, Cohort effects on prevalence. F, Cohort effects on YLDs. G, Local drifts for prevalence. H, Local drifts for YLDs. Abbreviations: YLDs, years lived with disability.

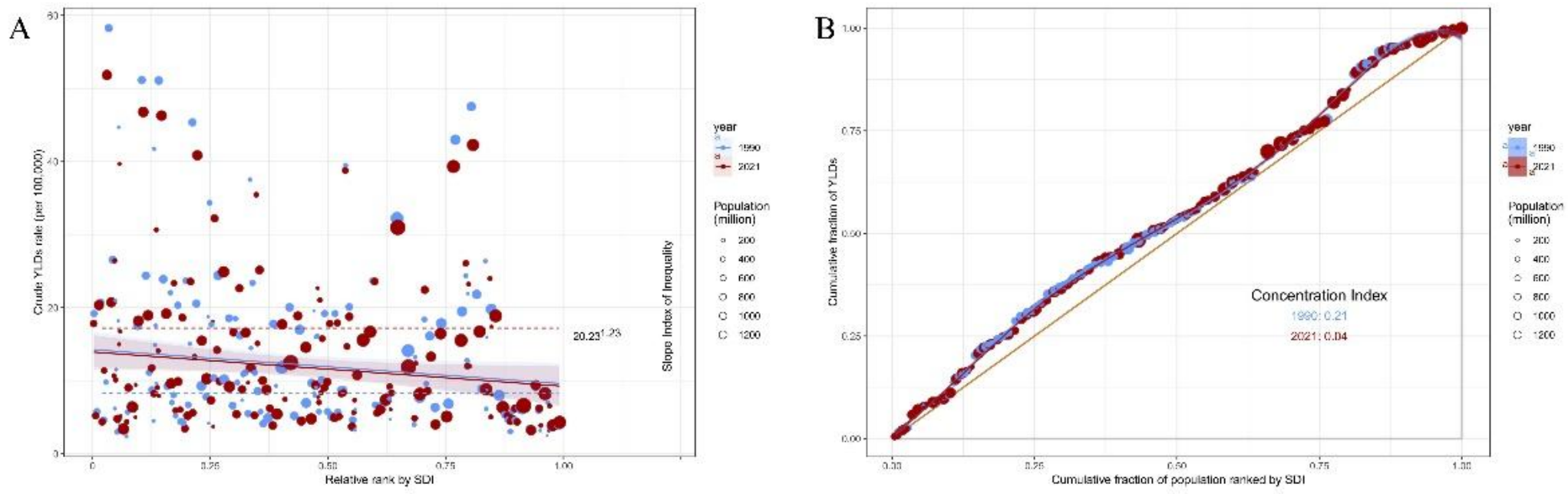

Supplemental Figure S4. Absolute and relative cross-country inequality in YLDs and ranks of ID from preterm birth from 1990 to 2021. A, Inequality regression curves for YLDs, 1990 and 2021. B, Concentration curves for YLDs, 1990 and 2021. Abbreviations: YLDs, years lived with disability; ID, intellectual disability.

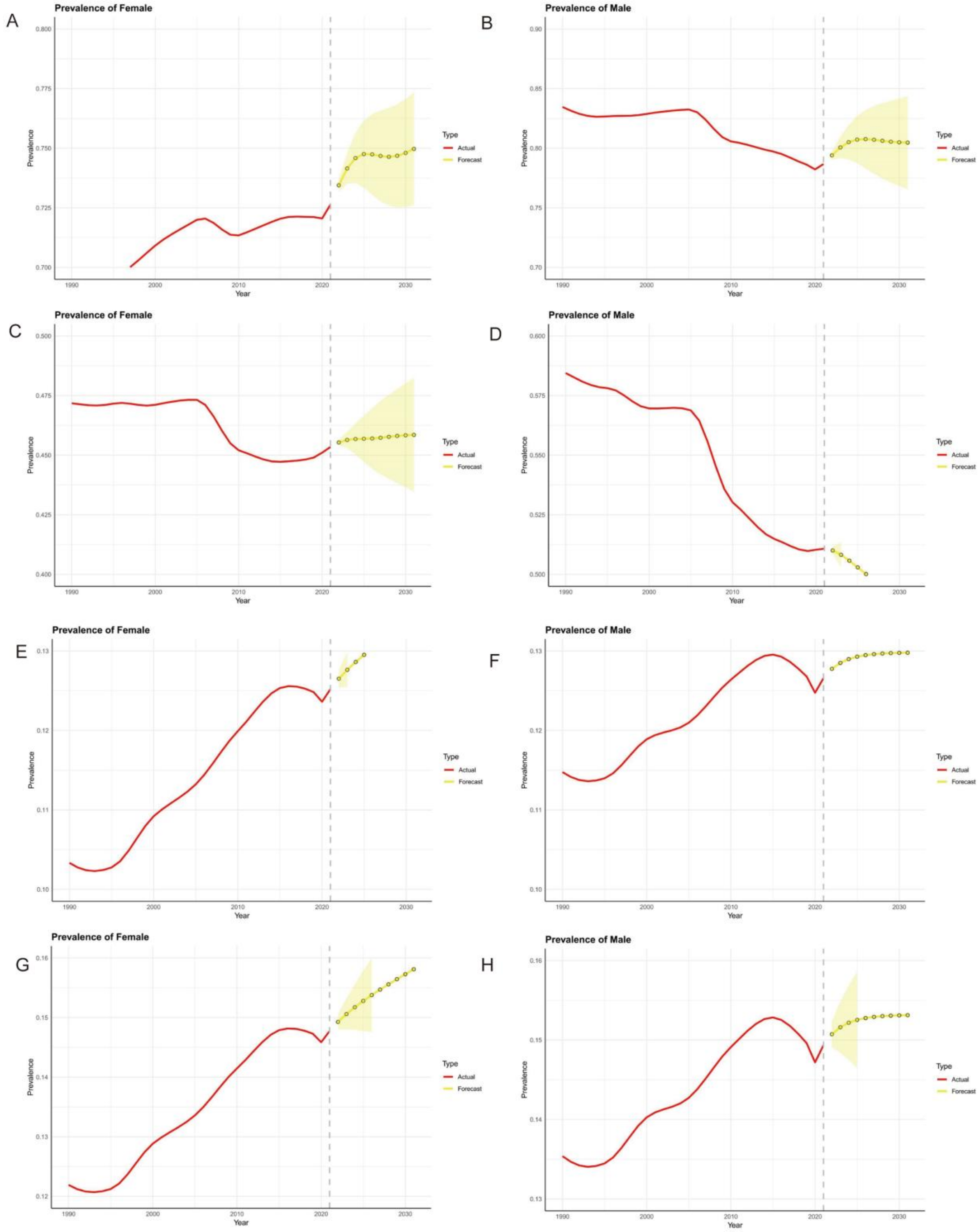

Supplemental Figure S5. ARIMA model predictions for prevalence on four IDs by premature birth from 2021 to 2031. A, ARIMA model predictions for prevalence on females developmental ID by premature birth from 2021 to 2031. B, ARIMA model predictions for prevalence on males developmental ID by premature birth from 2021 to 2031. C, ARIMA model predictions for prevalence on females mild ID by premature birth from 2021 to 2031. D, ARIMA model predictions for prevalence on males mild ID by premature birth from 2021 to 2031. E, ARIMA model predictions for prevalence

on females moderate ID by premature birth from 2021 to 2031. F, ARIMA model predictions for prevalence on males moderate ID by premature birth from 2021 to 2031. G, ARIMA model predictions for prevalence on females severe ID by premature birth from 2021 to 2031. H, ARIMA model predictions for prevalence on males severe ID by premature birth from 2021 to 2031. Abbreviations: ARIMA: Auto-Regressive Integrated Moving Average; ID, intellectual disability.

Table S1. Joinpoint trends of APCs and 95% CIs for each segment.

Abbreviations: APCs: annual percentage changes; CIs: confidence intervals

**Impairment**	**Gender**	**period**	**APC**	**95% CI**
Developmental ID	Male	1990-1993	-0.34	-0.58 - -0.11
		1993-2006	0.06	0.03 - 0.08
		2006-2009	-0.93	-1.41 - -0.45
		2009-2021	-0.26	-0.29 - -0.24
	Female	1990-1994	-0.11	-0.17 - -0.05
		1994-2005	0.36	0.34 - 0.37
		2005-2010	-0.22	-0.29 - -0.16
		2010-2016	0.20	0.15 - 0.25
		2016-2019	-0.07	-0.26 - 0.12
		2019-2021	0.35	0.17 - 0.54
Mild ID	Male	1990-1999	-0.25	-0.27 - -0.22
		1999-2006	-0.12	-0.17 - -0.07
		2006-2009	-1.92	-2.18 - -1.65
		2009-2014	-0.70	-0.78 - -0.61

		2014-2018	-0.30	-0.44 - -0.17
		2018-2021	0.02	-0.11 - 0.16
	Female	1990-2006	-0.25	-0.27 - -0.22
		2006-2009	-0.12	-0.17 - -0.07
		2009-2014	-1.92	-2.18 - -1.65
		2014-2019	-0.70	-0.78 - -0.61
		2019-2021	-0.30	-0.44 - -0.17
		1990-1999	0.02	-0.11 - 0.16
Moderate ID	Male	1990-1994	-0.21	-0.72 - 0.31
		1994-2015	0.65	0.61 - 0.69
		2015-2021	-0.63	-0.86 - -0.39
	Female	1990-1995	-0.12	-0.36 - 0.11
		1995-2000	1.32	1.01 - 1.62
		2000-2005	0.72	0.42 - 1.02
		2014-2021	1.13	1.03 - 1.24
Severe ID	Male	1990-1994	-0.21	-0.72 - 0.31
		1994-2015	0.65	0.61 - 0.69
		2015-2021	-0.63	-0.86 - -0.39
	Female	1990-1995	-0.12	-0.36 - 0.11
		1995-2000	1.32	1.01 - 1.62
		2000-2004	0.72	0.42 - 1.02
		2004-2015	1.13	1.03 - 1.24
		2015-2021	-0.07	-0.19 - 0.04
